# Association of Non-alcoholic Fatty Liver Disease with Metabolic Syndrome Independently of Central Obesity and Insulin Resistance

**DOI:** 10.1038/srep27034

**Published:** 2016-06-01

**Authors:** Kuen Cheh Yang, Hui-Fang Hung, Chia-Wen Lu, Hao-Hsiang Chang, Long-Teng Lee, Kuo-Chin Huang

**Affiliations:** 1 Department of Community and Family Medicine, National Taiwan University Hospital Hsinchu Branch, Hsinchu City, Taiwan; 2 Department of Family Medicine, National Taiwan University Hospital, Taipei, Taiwan; 3 Department of Family Medicine, National Taiwan University Hospital Bei-Hu Branch, Taipei, Taiwan; 4 Department of Family Medicine, College of Medicine, National Taiwan University, Taipei, Taiwan

## Abstract

Non-alcoholic fatty liver disease (NAFLD) is an emerging chronic liver disease that may lead to liver cirrhosis and hepatocellular carcinoma. We aimed to determine the association between the prevalence of metabolic syndrome (MetS) and NAFLD severity using semi-quantitative ultrasonography (US). A total of 614 participants were recruited from the community. NAFLD was evaluated according to the ultrasonographic Fatty Liver Indicator (US-FLI), which is a semi-quantitative liver ultrasound score. Insulin resistance was estimated with the homeostasis model assessment index for insulin resistance (HOMA-IR). NAFLD and MetS were found in 53.7 and 17.3% of the participants, respectively. Linear relationships were found between the severity of NAFLD and waist circumference, fasting glucose, HOMA-IR, triglycerides, HDL-C and blood pressure. After adjusting for confounding factors, i.e., body mass index and HOMA-IR, the odds ratios for MetS were 3.64 (95% confidence interval (CI): 1.5–8.83) for those with mild NAFLD and 9.4 (95% CI: 3.54–24.98) for those with moderate-to-severe NAFLD compared to those without NAFLD. The combination of the HOMA-IR and US-FLI scores better differentiated MetS than the HOMA-IR alone. In addition to obesity, the severity of NAFLD and the HOMA-IR both play important roles in MetS. Whether NAFLD is a component of MetS warrants further research.

Non-alcoholic fatty liver disease (NAFLD) includes a spectrum of diseases that range from simple steatosis to steatohepatitis, advanced fibrosis and cirrhosis. NAFLD is now the most common chronic liver disease in many developed countries^1^,^2^ and is closely associated with obesity and cardiovascular disease^3^,^4^. Furthermore, NAFLD is expected to become an even more serious public health issue because of the increasing prevalence of obesity and aging^5^,^6^,^7^. Metabolic syndrome (MetS) is a cluster of metabolic abnormalities that is a precursor to cardiovascular disease, and patients with NAFLD have a higher rate of MetS than those without NAFLD^2^,^8^,^9^,^10^. Moreover, NAFLD has also been reported to be independent of the traditional risk factors for subclinical atherosclerosis^11^,^12^, cardiovascular disease (CVD)^13^,^14^ and MetS^15^ and to increase the risk of mortality^16^,^17^. Both MetS and NAFLD involve interactions of adipokines, cytokines, inflammatory factors and insulin resistance, and some researchers have proposed that NAFLD can be regarded as a hepatic manifestation of MetS^9^,^18^. In contrast, some evidences have demonstrated that NAFLD dissociates from the features of MetS in familial hypobetalipoprotienemia (FHBL) and in subjects with a diacylglycerol acyltransferase 2 (DGAT2) gene polymorphism or patatin-like phospholipase 3 gene (PNPLA3) gene polymorphisms^19^. Taken together, it suggests that the association of NAFLD with MetS warrants more research.

Currently, a liver biopsy is the gold standard method for diagnosing NAFLD. Advanced imaging tools such as magnetic resonance spectroscopy (MRS) and computed tomography (CT), have also been used; however, they cannot be used in community surveys or epidemiological studies. Compared to invasive biopsy and expensive MRS and CT, ultrasonography (US) is relatively inexpensive and widely available in clinical settings, although it is unable to detect less than 10% steatosis of hepatocytes^20^. However, the majority of studies that have used US have defined NAFLD as a binary variable (i.e., normal vs. abnormal) and have not performed quantifications or gradings of liver fat. Therefore, there are limited data regarding whether US grades are associated with cardiometabolic risks in patients with NAFLD. A recent devised semi-quantitative scoring system has been demonstrated to mirror the severity of hepatic histological changes in NAFLD and the extent of steatosis^21^,^22^. Therefore, an ultrasonographic score may offer the potential to study and monitor liver fat in a community clinical setting.

Hepatic triacylglycerol and diacylglycerol (DAG) accumulation in livers with steatosis, in addition to protein kinase Cε (PKCε) activation, impairs hepatic insulin action. Therefore, NAFLD is strongly associated with hepatic insulin resistance^19^,^23^,^24^. The exacerbation of hepatic insulin resistance promotes the development and progression of cardiovascular disease and diabetes^4^,^25^. Notably, more hepatic fat is associated with a higher risk of CVD^13^,^15^. These findings imply that noninvasive techniques, such as MRS and liver US, with accurate measurements might be useful tools for assessing CVD risk and MetS^4^.

Therefore, the objective of this study was to investigate the associations between semi-quantitative US findings of NAFLD and the presence of MetS using a novel semi-quantitative ultrasonographic technique termed the Ultrasonographic Fatty Liver Indicator (US-FLI)^22^. Furthermore, we also assessed the relationships of NAFLD with insulin resistance and obesity in MetS.

## Methods

### Ethics statement

This study was performed in accordance with the principles of the Helsinki Declaration and Good Clinical Practice. The study protocol was approved by the Institutional Review Board of National Taiwan University Hospital (IRB NO. 201210012RIC). All of the subjects provided written informed consent.

### Subject enrollment

This study was conducted in the community of Hsinchu City. All of the participants completed detailed surveys that included standardized questionnaires. The exclusion criteria were excessive alcohol use (>20 g of alcohol daily for women and >30 g for men), and chronic liver diseases (including chronic hepatitis, autoimmune, drug-induced, vascular and inherited hemochromatosis and Wilson disease). The participants were evaluated for the presence of any exclusion criteria during recruitment via a questionnaire administered by our examiner. In total, 614 adults older than 20 years were enrolled.

Individual information about alcohol consumption, cigarette smoking, betel nut chewing, coffee intake, menstrual status, education level, physical exercise and sleeping conditions were obtained via a questionnaire administered by our examiner. Smoking status, alcohol consumption, betel nut chewing and coffee intake were defined as non-, current and previous. Current smokers and betel nut chewers were defined as those who had smoked tobacco or chewed betel nut for more than the past 6 months. Previous smokers and betel nuts chewers were defined as those who had quit for more than 1 year. Current alcohol drinkers were defined as those who drank more than one ounce of alcohol per week for 6 months. Previous drinkers were defined as those who had quit for more than 1 year. Current coffee intake was defined as drinking more than 1 cup of coffee (200 ml) per week for 6 months. Previous coffee intake was defined as those who had quit for more than 1 year. Menopause was defined as no menstruation for more than 1 year. Education level was classified as university or higher, junior or senior high school, and illiterate or elementary school. Physical activity was recorded as the number of hours of exercise performed per week. The sleeping condition was recorded as the number of hours slept per day. Diabetes, hypertension, and hyperlipidemia were defined based on a self-reported histories or current medication use for these conditions.

### Anthropometric indices and biochemical analyses

Anthropometric and metabolic data were collected by routine physical examinations. Body mass index (BMI) was recorded as the weight divided by height squared (kg/m^2^). Waist circumference (WC) was measured at the mid-level between the costal margins and the iliac crests. Fasting plasma glucose (FPG), total cholesterol (TCH), high-density lipoprotein (HDL-C), low-density lipoprotein (LDL-C), and triglycerides (TG) were measured after an 8-hour overnight fast. Insulin resistance was measured using the homeostasis model assessment index of insulin resistance (HOMA-IR)^26^ based on an iterative structural model that simulated physical processes and was developed by the Diabetes Trials Unit of the Oxford Center for Diabetes, Endocrinology, and Metabolism (http://www.dtu.ox.ac.uk/homacalculator).

### Ultrasonography assessment

Abdominal US scanning was performed after an 8-hour overnight fast by a well-trained examiner with a 3.5–5 MHz transducer and a high-resolution B-mode scanner (Hitachi Aloka ProSound α6). The ultrasound measurements were performed by three experienced research physicians with good professional backgrounds who were trained extensively in liver US. Before the study, all of the physicians reached a consensus regarding the standard procedure for ultrasound scanning, including the scoring of US-FLI and the sequence of acquiring liver images. All operators agreed on a standard procedure to follow during the examinations. A similar study of US-FLI has demonstrated a good inter-observer agreement (κ = 0.805–0.882, P < 0.001)^22^. The severity of NAFLD was calculated using the US-FLI score^22^, which ranges from 0 to 8. The US-FLI is composed of five indicators: (1) the presence of liver-kidney contrast graded as mild/moderate (score 2) and severe (score 3); and (2) the presences (score 1) or absences (score 0) of posterior attenuation of the ultrasound beam, vessel blurring, difficult visualization of the gallbladder wall, difficult visualization of the diaphragm and areas of focal sparing (score of 1 each). The subjects were then divided into three groups based on NAFLD severity according to the US-FLI score: normal (score 0–1), mild NAFLD (score 2–4), and moderate-to-severe NAFLD (score >4). In further analyses, we also divided the subjects into NAFLD quartiles according to the US-FLI scores as follows: 0–1, 2–3, 4–5, and ≥6.

### Definition of metabolic syndrome

The diagnostic criteria for MetS were based on the modified National Cholesterol Education Program Adult Treatment Panel III Criteria (NCEP-ATP III), for the Taiwanese population. The diagnosis required at least three of the following metabolic factors: 1) a WC of ≥90 cm for men or ≥80 cm for women; 2) a systolic blood pressure ≥130 mmHg, or a diastolic blood pressure ≥85 mmHg, or the use of medications for hypertension; 3) hyperglycemia (FPG ≥ 100 mg/dL) or the use of medications for diabetes; 4) hypertriglyceridemia (TG ≥ 150 mg/dL) or the use of medications for hyperlipidemia; and 5) low HDL-C (≤40 mg/dL in men and ≤50 mg/dL in women).

### Statistical analysis

The categorical data are presented as percentages, and the continuous variables are presented as the means ± the standard deviation. The categorical data were analyzed using the Chi-squared test. Comparisons of three or more groups were performed with analyses of variance (ANOVAs). The Cochran-Armitage trend test was used to test for trends in the anthropometric and metabolic factors using US-FLI score tertiles. The relationship between MetS and the severity of NAFLD was assessed using a multiple logistic regression model that included adjustments for age, gender, education level, alcohol consumption, smoking, betel nut chewing, coffee intake, hours of sleep, hours of exercise per week, menopause status (women only), BMI and HOMA-IR. The odds ratios (ORs) and 95% confidence intervals (CIs) were calculated. Univariate receiver operating characteristic (ROC) curves of the HOMA-IR and US-FLI scores were utilized to examine the ability to distinguish MetS. The areas under the ROC curves (AUCs) were used to compare the different univariate ROC models and joint models to HOMA-IR alone. Youden’s index was also used to identify the best cut-off value to diagnose MetS. The mean blood pressure was calculated as [2× diastolic blood pressure + systolic blood pressure]/3. The least squared means of WC, FPG, HOMA-IR, HDL-C and the mean blood pressure were computed using general linear models with adjustments for several confounders among the four US-FLI quartile groups. A P value < 0.05 was considered to be statistically significant. All of the statistical analyses were conducted using SAS version 9.3 (SAS Inc., Cary, NC, USA).

## Results

A total of 614 participants including 236 (38.4%) men and 378 (61.6%) women were recruited, and the participants’ average age was 42.6 ± 11.5 years. The overall prevalence of MetS was 17.6% and the average US-FLI score was 2.04 (Table 1). NAFLD was detected in 330 (53.7%) subjects, of whom 177 (28.8%) had mild steatosis and 105 (17.1%) had moderate-to-severe steatosis.

Table 2 presents the characteristics of the participants in the different US-FLI tertiles. There was no significant difference in age (P = 0.881). The percentage of men increased with the increasing US-FLI score (P < 0.0001). Compared to the subjects without NAFLD, those with higher US-FLI scores were heavier and exhibited greater BMI, total cholesterol, LDL-C, triglycerides, fasting glucose, HOMA-IR, percentage of MetS, abnormal liver function and body fat levels (P for trend: 0.0018 for TCHO; P for trend <0.0001 for the others). Since a US-FLI <4 has a high negative predictive value (94%) in ruling out the diagnosis of severe non-alcoholic steatohepatitis (NASH), we also investigated the differences between subjects with US-FLIs <4 and those US-FLIs ≥4. The metabolic factors of the subjects with US-FLIs ≥4 were significantly worse than those with US-FLIs <4 (Supplementary Table S1).

The associations between the severity of NAFLD and the prevalence of MetS from the multivariate logistic regression analyses are presented in Table 3. In model 1, a higher NAFLD level was correlated with a higher risk of MetS after adjusting for age, gender, education level, alcohol consumption, smoking, betel nut chewing, coffee intake, hours of sleep, hours of exercise per week and menopause status. The ORs for MetS for the second and third levels of NAFLD severity were 6.76 (95% CI: 3.18–14.37) and 35.5 (95% CI: 15.87–79.4), respectively, compared to those without NAFLD. In model 2, following a further adjustment for BMI, the ORs for MetS in the mild and moderate-to-severe NAFLD groups were 3.68 (95% CI: 1.67–8.11) and 9.84 (95% CI: 4.04–23.97), respectively, compared to those without NAFLD. In model 3, following a further adjustment for HOMA-IR, the ORs for MetS in the mild and moderate-to-severe NAFLD groups were 3.64 (95% CI: 1.5–8.83) and 9.4 (95% CI: 3.54–24.98), respectively, compared to those without NAFLD. When we replaced the US-FLI score with the severity of NAFLD in model 3, the OR b US-FLI was 1.39 (95% CI: 1.2–1.62, P < 0.0001).

Table 4 presents the AUC values for the US-FLI score and the HOMA-IR in the identification of individuals with MetS. The AUCs of HOMA-IR and US-FLI were 0.79 (95% CI: 0.75–0.84) and 0.8 (0.76–0.85), respectively. There was no significant difference between the HOMA-IR and US-FLI scores in terms of identifying MetS (P = 0.7422). However, the combination of US-FLI and HOMA-IR significantly improved the ability to distinguish MetS (P = 0.0131) compared to HOMA-IR alone.

Table 5 presents the diagnostic performance of the US-FLI score in the differentiation of MetS. The sensitivity of diagnosing MetS decreased with the increasing US-FLI score, whereas the specificity increased with the decreasing US-FLI score. According to Youden’s index, the best cut-off value for the US-FLI score for distinguishing MetS was 3, which yielded a sensitivity and a specificity of 0.77 (95% CI: 0.69–0.85) and 0.73 (95% CI: 0.69–0.77), respectively. The univariate ROC curve for US-FLI in the diagnosis of MetS can be found in Supplementary Fig. S1.

**Figure 1:**
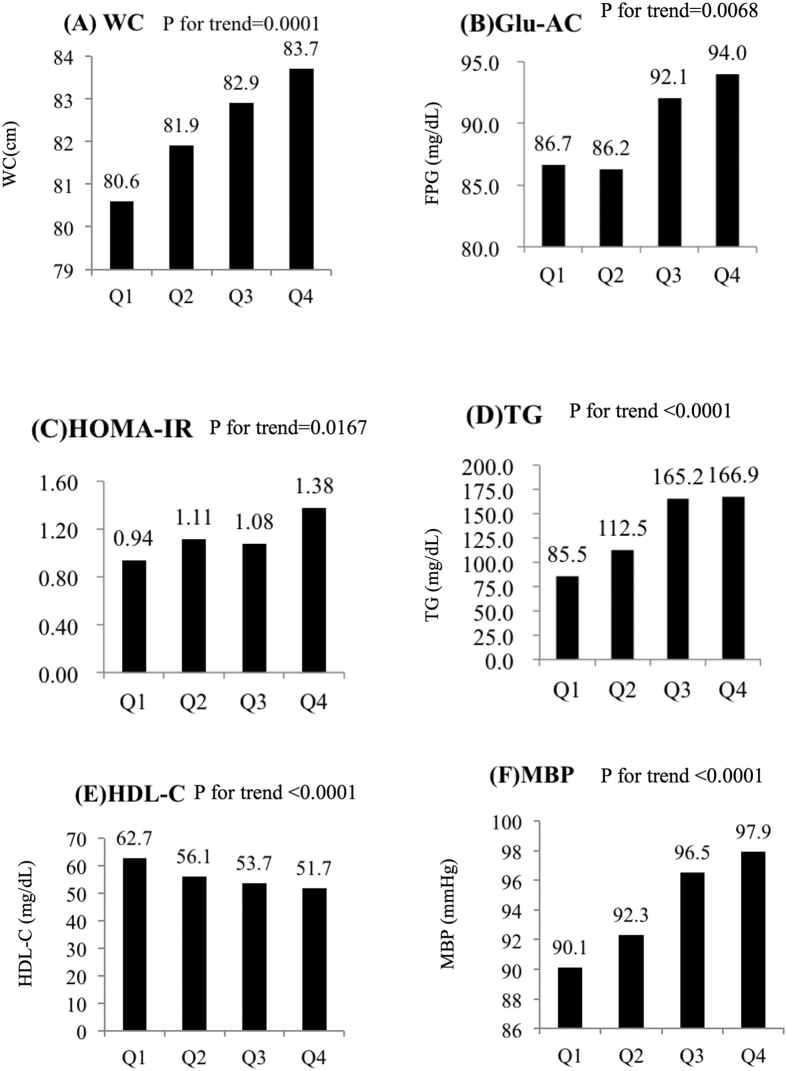
Comparisons of the (**A**) waist circumference (WC), (**B**) fasting plasma glucose (FPG), (**C**) HOMA-IR, (**D**) triglycerides (TG), (**E**) high-density lipoprotein cholesterol (HDL-C) and (**F**) mean blood pressure (MBP) parameters according to the ultrasonographic Fatty Liver Indicator (US-FLI) quartiles (i.e., the severity of NAFLD). The means ± SE were calculated with the least squared (LS) method using a multiple regression model after adjustments for age, gender, education level, betel nut chewing, alcohol consumption, smoking, menopause, coffee intake, hours of sleep, and hours of exercise per week. The LS means of (**A**–**F**) exhibited linear relationships with the severity of NAFLD (P for trend = 0.0001 for WC, P for trend = 0.0068 for fasting glucose, P for trend = 0.0167 for HOMA-IR, and P for trend <0.0001 for triglycerides, HDL-C, and MBP). Q1: US-FLI: 0–1; Q2: US-FLI: 2–3; Q3: US-FLI: 4–5; Q4: US-FLI ≥6.

Figure 1 presents the least squared means of WC, fasting glucose, HOMA-IR, triglycerides, HDL-C and mean blood pressure according to NAFLD severity. After adjusting for the confounding factors in model 3, all of the metabolic factors exhibited linear relationships with the severity of NAFLD (P = 0.0001 for WC, P = 0.0068 for fasting glucose, P = 0.0167 for HOMA-IR, and P < 0.0001 for triglycerides, HDL-C, and mean blood pressure).

## Discussion

To the best of our knowledge, this is the first study to demonstrate an association between increasing severity of NAFLD as assessed through the US-FLI score and a greater risk of MetS (OR per US-FLI score: 1.4, 95% CI: 1.2–1.6) after adjusting for BMI and insulin resistance. In accordance with our results, a previous study has demonstrated that non-esterified fatty acid concentrations are associated with NAFLD severity independently of insulin resistance^27^. Furthermore, we also observed significant relationships between the severity of NAFLD as assessed through the US-FLI score and metabolic factors. These results confirmed a previous study demonstrating that semi-quantitative liver US was able to detect metabolic derangements^22^.

Several diagnostic tools have been developed to predict hepatic steatosis using anthropometric parameters and blood biomarkers. However, these tools have only been validated in specific countries, or they have been suboptimal or the algorithm has not been published^28^. Currently, the best non-invasive tools for the diagnosis of NAFLD are imaging modalities. Although liver US has the disadvantage of a broad spectrum of sensitivity (range, 60–94%) for the diagnosis of NAFLD, it is widely used in a clinical settings and has been reported in many studies^20^. Compared to the subjective description of US images for NAFLD, a semi-quantitative scoring system for liver US provides a more objective evaluation and can improve the diagnosis of NAFLD^21^,^22^,^29^,^30^. Hamaguchi *et al*. reported an AUC of 0.98 for the diagnosis of NAFLD with a sensitivity of 91.7% in a Japanese cohort^21^. However, the histological evaluation was performed according to Matteoni’s classification, which was not used to identify the cases with steatosis only or steatosis and inflammation in the original study^31^. Saadeh *et al*. reported that their scoring system could only detect the severity of hepatic steatosis when the extent of fatty infiltration was >33%^30^. The scoring system proposed by Liang *et al*. was developed based on morbid obese patients^29^, and therefore it may not be applicable to the general population. Compared to Liang’s scoring system, the US-FLI has been reported to be more strongly and significantly positively correlated with the percentage of steatosis on histology (ρ = 0.745 vs. 0.528)^22^. Furthermore, US-FLI was developed according to Brunt’s diagnostic criteria, which are recommended for clinical purposes^32^,^33^. The inter-observer agreement reported for Saadeh’s score was only fair (κ = 0.4)^30^, that for Liang’s score was moderate/substantial (κ = 0.53–0.75)^29^, that for Hamaguchi’s score was almost perfect (κ = 0.95)^21^, and the score for US-FLI was substantial/almost perfect (κ = 0.805–0.882)^22^. Therefore, the US-FLI is more applicable, more easily calculated and better correlated with the histological parameters than the aforementioned scoring systems. Currently, the prevalence of NAFLD continues to increase in Asian populations^5^,^7^. However, semi-quantitative liver US scoring systems have not been applied extensively, which has limited studies exploring how liver content grading affects cardiometabolic risks. We demonstrated the ease of the application of US-FLI in Asian individuals for the first time in this study.

There was a very high prevalence of NAFLD in the current study; 53.7% of the patients had hepatic steatosis compared to 20–30% of adults in Western countries^14^. Different races and ethnicities have different liver fat partitioning, and this may play an important role^34^. Previous studies have demonstrated a variable range in the prevalence of NAFLD. A community study in central Taiwan reported an NAFLD prevalence of 11.5%^35^, and another study in southern Taiwan reported a much higher prevalence of 57.8%^36^. This difference may be partially attributable to the different liver US methods employed and the greater sensitivity of US-FLI.

Many cross-sectional studies have demonstrated that NAFLD is strongly associated with MetS^8^,^15^,^21^,^22^,^37^,^38^,^39^,^40^,^41^,^42^,^43^ or abnormal cardiometabolic factors^9^,^44^,^45^,^46^,^47^,^48^,^49^,^50^. Few prospective studies have indicated that NAFLD can predict a higher incidence of MetS^51^,^52^,^53^. A recent meta-analysis of prospective studies has demonstrated that NAFLD is associated with a nearly two-fold increased in the incident risk of type 2 diabetes and MetS. However, in the majority of studies, NAFLD was assessed according to abnormal liver enzyme^10^. Only a few studies have reported the use of CT^41^,^42^,^54^ or MRI^15^, and the majority of studies have used liver US to diagnose NAFLD. Among the studies that have used US, the diagnoses were based on the presence of ultrasonographic patterns consistent with a “bright liver”, such as brightness and posterior attenuation. Only one cross-sectional study^43^ and one prospective study in Korea^52^ have used a scoring system to diagnose NAFLD. Hamaguchi *et al*. reported that their semi-quantitative liver US scoring system was significantly correlated with MetS (OR: 1.37, 95% CI: 1.26–1.49)^21^, which is similar to our results (OR: 1.4, 95% CI: 1.2–1.6). However, these authors did not adjust for lifestyle factors or insulin resistance. Quantifications of the amount of liver fat in patients with NAFLD are strongly associated with metabolic factors, metabolic syndrome^15^,^52^,^55^ and cardiovascular disease^13^; however, the simple dichotomous classification of NAFLD (i.e., normal vs. abnormal) via liver US does not reflect the degree of hepatic steatosis. Because liver US is widely available, safe and inexpensive, it has been suggested as an acceptable first-line screening tool^56^. A recent study also revealed that the US fatty liver score exhibits an acceptable performance in the diagnosis of NAFLD (AUC: 0.82 [0.69–0.94]) and that this performance was statistically similar to that of liver ^1^H-MRS (AUC: 0.89 [0.41–0.89]. P = 0.15)^48^. Therefore, the standardization of semi-quantitative simple scoring systems may enable the prediction of metabolic abnormalities and liver histology changes^21^,^22^,^56^. Our results using US-FLI, which is a semi-quantitative liver US scoring system, revealed that such a system could illustrate the relationship between metabolic derangement and the severity of NAFLD.

NAFLD is not a currently a recognized component of MetS; however, NAFLD has been recommended as an additional criterion^38^,^57^. It is generally accepted that insulin resistance is the mechanism underlying MetS. One study with non-obese and non-diabetic Korean middle-aged adults demonstrated that individuals with NAFLD exhibited significantly higher insulin resistance than those without NAFLD, regardless of the number of abnormal metabolic factors. The sensitivity of NAFLD for insulin resistance has been reported to be higher than that for MetS (0.66 vs. 0.22)^39^. Pickhardt reported that the AUC for liver fat in the prediction of MetS was 0.706 (0.653–0.754) in a US population^58^. Our results extended these findings and demonstrated that compared to insulin resistance alone, the combination use of insulin resistance and NAFLD severity (US-FLI score) significantly improved the AUC from 0.79 (95% CI: 0.75–0.84) to 0.84 (95% CI: 0.8–0.88, P = 0.0131). Taken together, these findings support that notion that MetS and NAFLD predict the same risk profile. However, our study demonstrated that at the best cut-off point (i.e., an US-FLI score of 3) for MetS, the estimated probability of having MetS was only 0.19. Even with a US-FLI score of 8, the estimated probability of having MetS was 0.79. These findings are supported by previous findings that some but not all subjects with NAFLD and even some without NAFLD developed MetS^19^. These findings also imply that NAFLD might be an independent factor for MetS. A recent study using a confirmatory factor analysis of the US NHANES III database indicated that NAFLD is not an additional independent component of MetS^59^. However, Asian patients were not included in this study, and second-order confirmatory factor analysis should be applied to identify a better model fit. A large-scale bidirectional longitudinal cohort study in China that used Bayesian network causality demonstrated that the total effect of NAFLD on MetS was 2.49%, whereas the effect of MetS on NAFLD was 19.92%. However, the diagnosis of NAFLD was not subjected to any semi-quantitative indices, and the diagnosis of MetS was not based international criteria^60^. We also demonstrated the best cut-off value for the diagnosis of MetS (i.e., an US-FLI score of 3). The findings indicated that the sensitivity of the detection of MetS in individuals with US-FLI scores ≥3 was 77% (Table 4, Supplementary Fig. S1) and an individual with an US-FLI score ≥3 might comply with a “criterion” for MetS. Therefore, a simple, standardized liver US scoring system may assist in future research regarding whether the severity of NAFLD should be incorporated into the MetS criteria and whether it can be used clinically to diagnose MetS.

The liver is the site of the production of glucose and very-low-density lipoproteins (VLDLs) that contain the majority of triglycerides. This involvement means that MetS and NAFLD share the same risk profiles^61^. However, NAFLD can develop independently of insulin action in the liver. Increase in hepatic fat in insulin-resistant subjects arises because of dietary fat and adipocyte lipolysis without invoking insulin-driven *de novo* hepatic lipogenesis^23^,^62^. Hepatic fat is the most important predictor of hepatic insulin resistance^23^. Our study demonstrated that the severity of NAFLD was associated with MetS independently of HOMA-IR and obesity; moreover, it partly supports a crucial role of hepatic insulin resistance in determining the risk of CVD. The DAG-PKCε hypothesis of NAFLD has been validated in humans^23^,^25^,^63^. This theory emphasizes the important roles of hepatic fat and the dysregulation of lipid droplets that are shared by MetS and NAFLD. Recently, some studies have also highlighted the role of methylamines, which are the class of metabolites that are produced by intestinal microbiota. However, the exact mechanisms by which the microbiota contributing to MetS and NAFLD require additional research^64^.

There are some limitations to this study. First, we could not establish a temporal association between NAFLD and the occurrence of MetS because of the cross-sectional design. Second, we did not have data regarding sugar consumption, which is significantly associated with both MetS and NAFLD. Third, the participants in this study were not sampled systemically from the community. However, the participants were not significantly obese or thin, and the selection bias may have been small.

In conclusion, this is the first study to demonstrate that a semi-quantitative liver US scoring system, i.e., the US-FLI, can reflect the influence of NAFLD severity on MetS. In addition, the concept of NAFLD being a barometer of metabolic health^65^ was reinforced by this study results. We also linked insulin resistance and obesity to the association between the semi-quantitative liver US score and MetS. These findings support the notion that hepatic fat can induce metabolic abnormalities independently of obesity and insulin resistance^4^,^11^,^12^. More importantly, the results of the present study also suggest that US-FLI is a simple, cheap and accurate detector of the risk of MetS in Asian individuals. Because NAFLD will be an increasingly important public health issue in the future, further large-scale studies of NAFLD and metabolic disorders are warranted.

## Additional Information

**How to cite this article**: Yang, K. C. *et al*. Association of Non-alcoholic Fatty Liver Disease with Metabolic Syndrome Independently of Central Obesity and Insulin Resistance. *Sci. Rep.* **6**, 27034; doi: 10.1038/srep27034 (2016).

## Supplementary Material

Supplementary Information

## Figures and Tables

**Table 1:** Baseline characteristics of the participants.

Variable	N (%)	Mean ± SD	Range
Men	236 (38.4)		
Age (yrs)		42.6 ± 11.5	(19–79)
Menopause	46 (12.2)#		
MetS (%)	108 (17.6)		
Education level			
Illiterate or elementary	50 (8.2)		
Junior or senior high	164 (26.7)		
University or higher	400 (65.1)		
Smoking			
Never	520 (84.7)		
Current	68 (11.1)		
Previous	26 (4.2)		
Alcohol			
Never	496 (80.8)		
Current	105 (17.1)		
Previous	13 (2.1)		
Betel Nuts			
Never	586 (95.4)		
Current	28 (4.6)		
Coffee			
Never	276 (45)		
Current	338 (55)		
Sleeping ≥6 hrs	421 (68.6)		
Anthropometric variables			
Waist (cm)		81.1 ± 10.6	(55–120)
BMI (kg/m^2^)		24 ± 4.4	(14.9- 43)
Biochemistry parameters			
FPG (mg/dL)		88.1 ± 17.2	(58–272)
TCH (mg/dL)		195.8 ± 39.4	(101–320)
TG (mg/dL)		113.3 ± 88.1	(25–888)
HDL-C (mg/dL)		58.3 ± 15.6	(25–120)
LDL-C (mg/dL)		123.3 ± 33.3	(39–246)
AST (U/L)		22.7 ± 8.5	(11–68)
ALT (U/L)		25.7 ± 20.8	(2–151)
Insulin (μU/mL)		8.27 ± 7.17	(2–84.4)
HOMA-IR		1.06 ± 0.9	(0.25–10.2)
**NAFLD parameters**			
US-FLI Score		2.04 ± 2.19	(0–8)
Mild level (US-FLI Score ≥2)	330 (53.7)		
Moderate-and-severe level (US-FLI Score >4)	105 (17.1)		

Abbreviations: FPG: fasting glucose; TCH: total cholesterol; TG: triglycerides; HDL-C: high-density lipoprotein cholesterol; LDL-C: low-density lipoprotein cholesterol; AST: aspartate aminotransferase; ALT: alanine aminotransferase; HOMA-IR: homeostasis model assessment index; NAFLD: non-alcoholic fatty liver disease; US-FLI score: ultrasonographic Fatty Liver Indicator score.

^#^Percentage of women.

**Table 2:** Characteristics according to NAFLD severity$.

	Normal	Mild	Moderate-to- Severe	
	N = 284	N = 225	N = 105	P for trend
Male (%)	74 (26.1%)	98 (43.6%)	64 (61%)	<0.0001
Age(yrs)	41.7 ± 11.2	44.3 ± 12.3	41.6 ± 10.3	0.881
Waist (cm)	75.3 ± 7.8	83.3 ± 8.9	93.5 ± 8.6	<0.0001
BMI (kg/m^2^)	21.6 ± 2.8	24.7 ± 3.6	28.7 ± 4.3	<0.0001
FPG (mg/dL)	84.3 ± 12.5	88.3 ± 13.6	97.8 ± 28.1	<0.0001
TCH (mg/dL)	190.9 ± 32.8	198.7 ± 37.4	203 ± 36.1	0.0018
TG (mg/dL)	78.4 ± 39.3	123.9 ± 86.6	185.2 ± 128.7	<0.0001
HDL-C (mg/dL)	65.4 ± 15	55.1 ± 13.6	46.3 ± 10.3	<0.0001
LDL-C (mg/dL)	116.1 ± 30.8	127.9 ± 34.8	133.2 ± 32.4	<0.0001
MetS (%)	9 (3.2%)	45 (20%)	54 (51.4%)	<0.0001
Insulin (μU/mL)	5.69 ± 4.2	8.92 ± 8.42	12.38 ± 7.17	<0.0001
HOMA2-IR	0.73 ± 0.55	1.14 ± 1.04	1.61 ± 0.92	<0.0001
Body fat (%)	26.3 ± 6.9	29.5 ± 8.1	32.8 ± 7.9	<0.0001
Abnormal liver function#	12 (4.2%)	43 (19.1%)	53 (50.5%)	<0.0001

Abbreviations: BMI: body mass index; FPG: fasting plasma glucose; TCH: total cholesterol; TG: triglycerides; HDL-C: high-density lipoprotein cholesterol; LDL-C: low-density lipoprotein cholesterol; MetS: metabolic syndrome; HOMA-IR: homeostasis model assessment index; US-FLI: ultrasonographic Fatty Liver Indicator.

^$^Normal: US-FLI = 0–1; mild: US-FLI: 2–4, moderate-to-severe: >4.

^#^Abnormal liver function was defined by alanine aminotransferase (ALT) ≥41 U/L in men and ≥31 U/liter in women).

**Table 3:** Odds ratio (OR) for the non-alcoholic fatty liver severity level for metabolic syndrome^*^.

	Severity of non-alcoholic fatty liver disease (NAFLD)			
	Normal (N = 284)	Mild (N = 255)	Moderate-to-Severe (N = 105)	P-Value of NAFLD
	US-FLI <2	US-FLI: 2–4	US-FLI: >4	
Model 1	1	6.76 (3.18–14.37)	35.5 (15.87–79.4)	<0.0001
Model 2	1	3.68 (1.67–8.11)	9.84 (4.04–23.97)	<0.0001
Model 3#	1	3.64 (1.5–8.83)	9.4 (3.54–24.98)	<0.0001

Model 1: adjusted for age, gender, smoking, alcohol, betel nuts, exercise time per week and menopause (women only).

Model 2: model 1 plus further adjustment for BMI; odds ratio (OR) for BMI: 1.25 (95% confidence interval [CI]: 1.16–1.35, P-value < 0.0001).

Model 3: model 2 plus further adjustment for HOMA-IR; OR for BMI: 1.19 (95% CI: 1.1–1.3, P-value < 0.0001); OR for HOMA-IR: 1.87 (95% CI: 1.34–2.59, P-value = 0.0002).

^#^The NAFLD severity was replaced with the US-FLI score. OR for US-FLI: 1.39 (95% CI: 1.2–1.62, P < 0.0001).

**Table 4:** The area under the receiver operating characteristic (ROC) curve for metabolic syndrome.

	AUC	95% CI of AUC	P-value#
Model$	0.841	0.8–0.88	0.0131
HOMA-IR	0.794	0.746–0.843	Reference
US-FLI	0.8	0.759–0.848	0.7422

Abbreviations: AUC: area under the ROC curve; HOMA-IR: homeostatic model assessment for insulin resistance; US-FLI: ultrasonographic Fatty Liver Indicator.

^$^Using HOMA-IR and US-FLI to differentiate the metabolic syndrome.

^#^The AUC for each variable and that of the entire model were compared to the HOMA-IR alone.

**Table 5:** Diagnostic performances of the US-FLI score thresholds for predicting metabolic syndrome (MetS)*.

US-FLI Score	Estimated Probability of MetS	Sensitivity (mean ± 95% CI)	Specificity (mean ± 95% CI)
0	0.04	1	0
1	0.07	0.94 (0.9–0.99)	0.49 (0.45–0.54)
2	0.12	0.92 (0.86–0.97)	0.54 (0.5–0.59)
3	0.19	0.77 (0.69–0.85)	0.73 (0.69–0.77)
4	0.29	0.63 (0.54–0.72)	0.83 (0.8–0.86)
5	0.42	0.50 (0.41–0.59)	0.9 (0.87–0.93)
6	0.55	0.32 (0.24–0.41)	0.95 (0.93–0.97)
7	0.68	0.11 (0.05–0.17)	0.98 (0.97–0.99)
8	0.79	0.02 (0–0.04)	0.99 (0.99–100)

Abbreviations: US-FLI: ultrasonographic Fatty Liver Indicator; CI: confidence interval.

^*^US-FLI = 3 was the best cut-off point for diagnosing MetS according to the Youden index.
