# Supplementary material for: Association of Non-alcoholic Fatty Liver Disease with Metabolic Syndrome Independently of Central Obesity and Insulin Resistance
Source: Sci Rep. 2016 Jun 1;6:27034. doi: 10.1038/srep27034 (PMC4887873; doi:10.1038/srep27034)
Supplement: Supplementary Information [file srep27034-s1.pdf]

Association of Non-alcoholic Fatty Liver Disease with Metabolic Syndrome Independently of Central Obesity and Insulin Resistance

Kuen Cheh Yang[1,2], Hui-Fang Hung[1,2], Chia-Wen Lu[2], Hao-Hsiang Chang[2], Long-Teng Lee[2], Kuo-Chin Huang[2,3,4]

[1]Department of Community and Family Medicine, National Taiwan University Hospital Hsinchu Branch, Hsinchu City, Taiwan

[2]Department of Family Medicine, National Taiwan University Hospital, Taipei, Taiwan

[3]Department of Internal Medicine, National Taiwan University Hospital Bei-Hu Branch, Taipei, Taiwan

[4]Department of Family Medicine, College of Medicine, National Taiwan University, Taipei, Taiwan

Supplementary Table S1: Characteristics of the patients with US-FLIs < 4 and US-FLIs ≥ 4

	US-FLI < 4<br>N = 284	US-FLI ≥ 4<br>N = 225	P value[$]
Male (%)	154 (33.4%)	82 (53.6%)	< 0.0001
Age (yrs)	42.4 ± 11.6	43.2 ± 11.1	0.881
Waist (cm)	77.6 ± 8.4	92.1 ± 9.2	< 0.0001
BMI (kg/m^2)	22.5 ± 3.2	28.3 ± 4.2	< 0.0001
FPG (mg/dL)	85.2 ± 11.5	96.8 ± 26.3	< 0.0001
TCHO (mg/dL)	192.7 ± 34.7	205.3 ± 36.1	0.0001
TG (mg/dL)	91.8 ± 55.7	178.2 ± 157.5	< 0.0001
HDL-C (mg/dL)	61.6 ±15.5	58.4 ± 10.9	< 0.0001
LDL-C (mg/dL)	119.6 ± 32.5	134.7 ± 33.2	< 0.0001
MetS (%)	40 (8.7%)	68 (44.4%)	< 0.0001
Insulin (µU/mL)	6.89 ± 6.82	11.81 ± 6.84	< 0.0001
HOMA2-IR	0.88 ± 0.85	1.54 ± 0.88	< 0.0001
Body fat (%)	27.1 ± 7	33.2 ± 8.6	< 0.0001
Abnormal liver function	40 (8.7%)	68 (44.4%)	< 0.0001

[$] Student's t tests were used for the continuous variables, and Chi-square tests were used for the categorical variables.

Abbreviations: BMI, body mass index; FPG, fasting plasma glucose; TCH: total cholesterol; TG: triglycerides; HDL-C: high-density-lipoprotein cholesterol; LDL-C: low-density lipoprotein cholesterol; MetS: metabolic syndrome; HOMA-IR: homeostasis model assessment index.

Supplementary Figure S1: Univariate ROC curve for US-FLI in the diagnosis of MetS

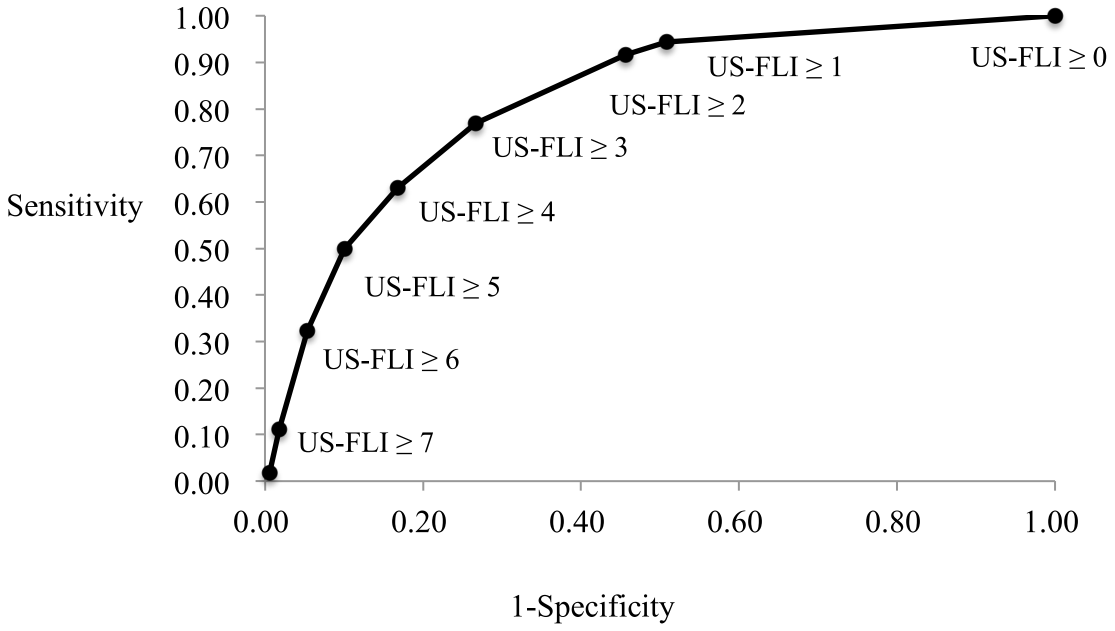

Abbreviations: ROC: receiver operating characteristic; MetS: metabolic syndrome.
